# The Role of Omentin-1 in Cancers Development and Progression

**DOI:** 10.3390/cancers15153797

**Published:** 2023-07-26

**Authors:** Paweł Dec, Agata Poniewierska-Baran, Andrzej Modrzejewski, Andrzej Pawlik

**Affiliations:** 1 Department of Plastic and Reconstructive Surgery, 109 Military Hospital, 71-422 Szczecin, Poland; pawel_dec@onet.pl; 2 Institute of Biology, University of Szczecin, 71-412 Szczecin, Poland; agata.poniewierska-baran@usz.edu.pl; 3 Department of Physiology, Pomeranian Medical University, 70-111 Szczecin, Poland; 4 Department of Surgery, Pomeranian Medical University, 71-422 Szczecin, Poland; amodrzejewski@interia.pl

**Keywords:** omentin-1, intelectin-1, cancer, carcinogenesis, adipokines, visceral adipose tissue

## Abstract

**Simple Summary:**

Adipocytes associated with visceral adipose tissue release omentin-1 (also known as intelectin-1), an adipokine whose gene is located at the chromosomal region 1q22–q23. This adipokine has been linked to type 2 diabetes in several populations. Omentin-1 is an anti-inflammatory and pro-apoptotic adipokine that helps adipocytes communicate with surrounding tissues to regulate glucose and fat metabolism. Higher omentin-1 expression has been reported in a number of malignancies, including liver cancer, prostate cancer, colon and colorectal cancer, gastric cancer, pancreatic adenocarcinoma, and breast cancer. This review discusses the role of omentin-1 in the process of cancer development and progression.

**Abstract:**

Adipose tissue serves as an energy store and is also an active endocrine organ, exerting activity that influences obesity-related processes through the production of regulatory proteins called adipokines or adipocytokines. Adipokines play important direct and indirect roles in the pathogenesis of insulin resistance, the regulation of local and systemic inflammatory processes, and related metabolic complications. There have been an increasing number of studies showing the relationship between some adipokines and carcinogenesis. This work reviews the current literature concerning the effects of omentin-1 on carcinogenesis.

## 1. Introduction

Cancers are the second most common cause of death worldwide [1]. Numerous risk factors for cancer development are currently being explored. In recent years, it has been noted that obesity, and thus excess body fat, is a significant risk factor for certain cancers, including colorectal, gallbladder, oesophageal, gastric, pancreatic, breast, endometrial, ovarian, and thyroid cancers [2,3,4,5,6,7,8,9]. Excessive adipose tissue, especially visceral adipose tissue, causes the development of chronic inflammation, leading to the development of numerous metabolic disorders, immune system dysfunction, and increased oxidative stress with the formation of free radicals [5,6,7]. This causes changes in the proteins produced and the synthesis of angiogenic factors, as well as the formation of mutations in DNA and changes at the epigenetic level that lead to neoplastic transformation. Other obesity-related factors that can lead to cancer include insulin resistance; hyperinsulinemia; and the abnormal synthesis of some cytokines, chemokines, hormones, and adipokines [10].

Adipose tissue is an important endocrine organ: it secretes adipokines that regulate several physiological processes and influence numerous pathological processes, including disorders of the immune system, the development of chronic inflammation, disorders of angiogenesis and haematopoiesis, and the process of carcinogenesis. Adipokines exert pleiotropic effects: they can both inhibit the process of carcinogenesis and exacerbate it [11]. Visceral adipose tissue, which is the source of many adipokines, has the greatest secretory role. It has a different metabolic profile than subcutaneous adipose tissue. It contains more inflammatory cells, especially macrophages, which secrete numerous inflammatory mediators and growth factors [12]. Under the influence of fatty acids secreted by adipocytes, immune cells are activated, leading to a further increase in inflammation. There is also an increase in oxidative stress and hypoxia in adipocytes, which leads to the production of hypoxia-induced transcription factors and the secretion of numerous chemotactic factors that increase macrophage activation and adipokine production [13].

Chronic inflammation, increased levels of oxidative stress, and activation of transcription factors lead to the induction of carcinogenesis [14]. Numerous adipokines are involved in this process and exhibit multidirectional effects. These processes have so far been poorly understood. Both increased and decreased concentrations of individual adipokines have been observed in various cancers. Studies suggest that adipokines may influence the course of carcinogenesis and serve as biomarkers of its development. Moreover, attempts have been made to use adipokines in anti-cancer therapy [15,16,17]. Newly discovered adipokines appear to be important markers of cancer progression. Understanding the role of adipokines in the process of carcinogenesis may be helpful in better understanding it and, consequently, in developing effective therapies [18,19,20]. One adipokine whose involvement is being considered in the development of the cancer process is omentin-1.

## 2. Characteristics of Omentin-1

Omentin-1—also known as intelectin-1 (ITLN-1), galactofuranose-binding lectin, or intestinal lactoferrin receptor—is found mainly in visceral adipose tissue (lattice and epicardial), mesothelial cells, vascular cells, airway cup cells, the small intestine, the colon, ovaries, as well as blood plasma. The major problem of the modern world is metabolic disorders, like obesity or type 2 diabetes mellitus, related to insulin resistance. Omentin increases insulin sensitivity by activating insulin-dependent glucose uptake in visceral and subcutaneous adipose tissue cells, as well as in muscles and the liver [21,22].

Work on the concentration of omentin in the serum of people with metabolic syndrome (MS) was carried out by Bremer et al. [23]. They described a reduced concentration of omentin in the serum and subcutaneous adipose tissue in patients with metabolic syndrome (MS) in relation to the control group, regardless of obesity. As demonstrated by Barth et al., the plasma concentration and mRNA expression of omentin in adipose tissue was decreased in obese people [21,24]. Similar results that omentin-1 concentrations decrease with increasing body weight, BMI, and body fat content were shown by others [25,26,27]. The concentration of omentin-1 had negative correlations with body mass index (BMI), fasting blood glucose level, the insulin resistance index HOMA-IR (homeostasis model assessment of insulin resistance), waist circumference, and insulin resistance. In addition, positive correlations of omentin-1 levels were found with high-density lipoprotein (HDL cholesterol) and plasma adiponectin concentrations [28].

The omentin-1 concentration in human blood may have a wide range of concentrations, from several ng/mL up to 300–600 ng/mL in healthy individuals, and women usually have higher plasma and serum concentrations of this adipokine compared to men. The plasma level of omentin-1 is negatively associated not only with diabetes but its complications as well. A 2021 case–control study showed that patients with diabetes had significantly lower omentin levels in comparison to patients without diabetes, but also that its level was significantly lower in patients with complications than in those without [29]. Serum omentin-1 can be used as a biomarker for obesity-related metabolic disorders because, according to the research, omentin-1 is inversely related to obesity, insulin resistance, and systolic blood pressure (SBP) [30]. Low or high levels of omentin can be an indicator of the advancement of cancer, depending on the type. Some data showed that the development of cancer is associated with an increased amount of omentin in the blood serum, e.g., clinical data showed that the level of plasma omentin-1 is highly expressed in patients with colorectal cancer, so it can be an independent risk factor for the recurrence and survival of patients [31]. According to other data, its concentration may decrease with the malignancy of the cancer [32]. Interestingly, significant differences were observed in the concentrations of omentin-1, depending on normal BMI or higher BMI, which is described in more detail in the following chapters [33,34,35]. It should therefore be noted that the increase or decrease in serum omentin depends not only on the presence of cancer, its type, and advancement, but also on the health status of patients (BMI, comorbidities, amount of adipose tissue, etc.).

Omentin-1 is mostly secreted from visceral adipose tissue (approximately 20-fold higher) compared with subcutaneous adipose tissue [28]. Omentin-1 expression in preadipocytes is reduced by glucose/insulin, while it is stimulated by fibroblast growth factor 21 and dexamethasone. In the absence of insulin, omentin-1 enhances the phosphorylation of Akt, a serine-threonine kinase, which can affect cell proliferation and metabolism and inhibit apoptosis.

There are two isoforms of omentin: omentin-1 and omentin-2. Omentin-2 shares 83% amino acid identity with omentin-1. The genes for both isoforms are located side by side at the chromosomal region 1q22–q23 [28], which has been linked to type 2 diabetes in several populations. Numerous studies have shown the important influence omentin-1 plays on the body’s metabolic processes, including insulin sensitivity and anti-inflammatory, anti-atherosclerotic, and cardiovascular protective effects. Omentin-1 consists of 313 amino acids with a molecular weight of about 35 kDa; it contains a 16-amino acid N-terminal signal peptide. In its structure, one can distinguish a fibrinogen-like domain (amino acids at positions 38–82) and a lectin-like domain (positions 37–313). In its native form, it exists as a glycosylated trimer with a molecular weight of about 120 kDa [36]. Plasma omentin-1 concentrations are elevated in many cancers, including malignant pleural mesothelioma (MPM) [37], liver cancer [19], prostate cancer [38], colon and colorectal cancer [39,40], gastric cancer [41], and pancreatic adenocarcinoma [42]. The opposite results have been observed in renal cell carcinoma, in which omentin-1 levels are dramatically reduced [32].

Omentin-1 reduces the expression of the adhesion proteins vascular cell adhesion molecule-1 (VCAM-1) and intercellular cell adhesion molecule-1 (ICAM-1) by inhibiting the p38, c-Jun N-terminal kinase (JNK) and extracellular signal-regulated kinase(ERK)/nuclear transcription factor kappa B (NF-κB) signaling pathways [43,44,45]. In cells under the influence of omentin-1, the Bax/Bcl-2 protein ratio increases, resulting in the activation of the caspase-3 signaling pathway. Further investigation of the mechanism showed that omentin-1 increases p53 protein levels by decreasing p53 deacetylation, thereby increasing p53 protein stability [46].

The increase in omentin-1 levels described in some cancers (e.g., prostate, colon) is a part of the body’s defence mechanism against cancer cells. Some authors suggest that omentin-1 may exert a stimulatory effect on cancer progression via the Akt signaling pathway. It is suspected that omentin-1 has a pivotal role in carcinogenesis by increasing the secretion of angiogenic cytokines to affect blood vessel permeability and regeneration [45]. In an experimental study in a mouse model, omentin-1 increased Bcl-2 activity and reduced Bax in mesenchymal stem cells after incubation. There was inhibition of mitochondrial apoptosis with inhibition of caspase-3 and preservation of mitochondrial membrane potential. Omentin-1 could increase the secretion of angiogenic growth factor and enhance the ability of mesenchymal stem cells to stimulate umbilical vein endothelial cells (HUVEC). In addition, omentin-1 enhances Akt phosphorylation; however, blockade of the phosphoinositide-3-kinase (PI3K)/Akt pathway with the inhibitor LY294002 suppressed the above beneficial effects of omentin-1 [46].

Some omentin genetic variations may change insulin metabolism and have key roles in developing type 2 diabetes (T2D) through insulin resistance. As described by Khosi et al., there are significant differences between omentin Val109Asp and FTO rs9939609 polymorphisms in studied individuals. These genetic polymorphisms are significantly related to a higher HOMA index, but only Val109Asp polymorphism was related to overweight/obese individuals. They showed that both polymorphisms were positively correlated to familial history of diabetes [47]. Suliga et al. investigated the possible association between omentin-1 rs2274907 polymorphism and the risk of metabolic syndrome, but there were no associations of this polymorphism with any of the MetS components (abdominal obesity, increased TG and glucose concentration, decreased HDL-cholesterol concentration, and elevated blood pressure) [48]. As shown, the AT genotype of omentin-1 rs2274907 A/T polymorphism may be associated with increased BMI but not associated with T2D risk [49].

The effect of omentin gene polymorphism on cancer disease development has been studied. In 2021, a case–control study showed that rs2274907 A > T is a promising biomarker for CRC prognosis because the interaction between the rs2274907 A allele and BMI increased the CRC risk [50]. Individuals with the rs2274907 A > T AA genotype had similar tumor localizations, sizes, histological grades, and N and M stages to T allele carriers but presented with significantly higher T stages and TNM stages. In addition, serum omentin-1 levels are increased in patients with a more aggressive disease burden based on the Elston–Ellis grading system [51]. A study of Val109Asp omentin gene polymorphism showed that the Val allele increased the risk of breast cancer, as compared to the Asp allele [52].

## 3. The Role of Omentin-1 in Selected Cancers

**Figure 1:**
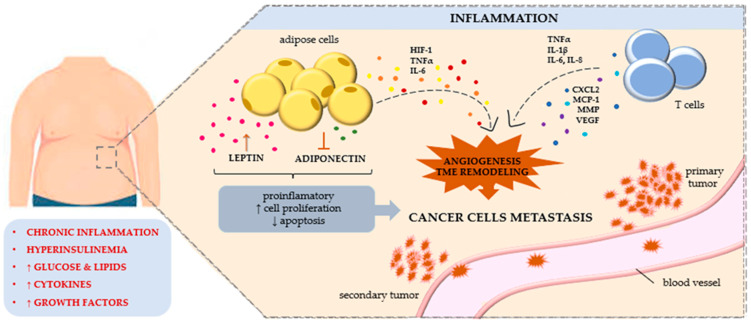
The crosstalk between adipose tissue and carcinogenesis. Abbreviations: HIF-1, hypoxia-inducible factor 1; TME, tumor microenvironment; TNFα, tumor necrosis factor α; IL, interleukin; CXCL2, CXC motif chemokine ligand 2; MCP-1, monocyte chemoattractant protein-1; MMP, matrix metalloproteinases; VEGF, vascular endothelial growth factor.

**Figure 2:**
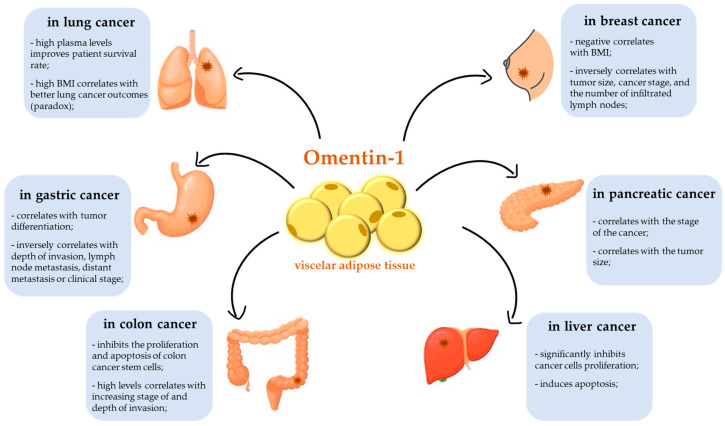
Influence of omentin-1 on carcinogenesis. Abbreviations: BMI, body mass index.

Adipose tissue is an active endocrine organ. Adipose tissue (composed of adipocytes) communicates with other organs and systems by releasing numerous bioactive proteins and adipokines, which affect the pathogenesis of obesity-linked diseases, including cancers. The link between obesity and cancer has been recognized for years. Human omentin-1 was isolated for the first time in 2001 from a small intestine complementary DNA (cDNA) library [53]. Since then, a series of studies have indicated the potential roles of omentin-1 in tumourigenesis. Omentin-1 has an important role in the proliferation and regulation of apoptosis in many types of cancer cells (Figure 1). At the same time, omentin-1 can increase the permeability of blood vessels and promote the metastasis of cancer cells, changes that shorten the survival time of patients. Because omentin-1 is secreted from the visceral fat accumulated in the omentum, it seems that omentin-1 should be crucial for the metastasis of cancers that penetrate the abdominal cavity. Until very recently, the individual adipokine profile (omentin-1 and other adipokines) of patients with cancers had practically been ignored: it had not been considered in the prognosis and staging of the disease. Currently, more and more scientists and physicians associate the relationship between the concentration and expression of adipokines, including omentin-1, with the prognosis, treatment effectiveness, and chance for a longer life for patients with cancer, as shown in Figure 2.

### 3.1. Colorectal Cancer

Adipose tissue metabolism plays an important role in the pathogenesis of colorectal cancer due to the secretion of pro-inflammatory cytokines, growth factors, and hormones. It is also believed that insulin resistance, glucose intolerance, elevated plasma insulin levels, body mass index (BMI), insulin-like growth factor (IGF-1), serum glucose, and free fatty acids are associated with the pathogenesis of colorectal cancer [34]. There have been several recent studies showing that plasma omentin-1 levels are elevated in patients with colorectal cancer and that high levels of omentin-1 may be a prognostic marker closely associated with future colorectal cancer risk. Zhao et al. [54] reported significantly higher mean omentin-1 levels in Chinese patients with colorectal cancer compared with the control group. Patients with higher omentin-1 levels had an increased risk of colorectal cancer regardless of age; BMI; waist-to-hip ratio (WHR); HOMA-IR; cholesterol and triglyceride concentrations; lifestyle; medications taken; family history of colorectal cancer or diabetes; and levels of adiponectin, leptin, visfatin, and resistin. Importantly, the authors observed an association between high levels of omentin-1 and increasing stage of colorectal adenocarcinoma and depth of invasion [54]. However, the results of other studies are inconsistent. Kim et al. [55] found omentin-1 to be a marker with a positive predictive significance of outcome in stage IV colorectal cancer, indicating that omentin-1 acts as a tumor suppressor in gastrointestinal cancer. In contrast, Aleksandrova et al. [56] reported that in a large prospective cohort study, higher omentin-1 levels were associated with a higher risk of colorectal cancer. The results of an immunohistochemical investigation by Zhang et al. [57] showed that the increase in omentin-1 expression was mainly localized in the cytoplasm of colon cancer cells. Omentin-1 protein and messenger RNA (mRNA) expression was higher in colon cancer tissues compared with surrounding tissues with normal morphology. In in vitro experiments, the authors found that omentin-1 affects SW480 cells—a colon cancer cell line—via autocrine and endocrine mechanisms.

Uyeturk et al. [41] found significantly elevated omentin-1 levels in patients with stage III colorectal cancer treated with surgery and adjuvant chemotherapy with oxaliplatin and 5-fluorouracil, compared with healthy controls who did not have surgery or chemotherapy. In a study of colon cancer cell cultures, Maeda et al. [58] observed a relationship between the transmembrane protein TMEM207 and omentin-1 production. Inhibition of TMEM207, which is mediated by a small interfering RNA, increased polyubiquitination and proteasomal degradation of the omentin-1 in cultured cells and thus decreased omentin-1 secretion. According to the authors, decreased levels of omentin-1 can lead to a poor prognosis in patients with advanced colorectal cancer [58]. Similarly, Kawashima et al. [20] showed that blockade of TMEM207, which is involved in omentin-1 processing, results in insufficient production of omentin-1, thus promoting colon carcinogenesis. The omentin-1/TMEM207 axis may play a role as a prognostic biomarker of colon cancer in the future.

Ji et al. [59] isolated CD133+ colon cancer stem cells from SW480 cells. CD133+ colon cancer stem cells obtained by indirect immunomagnetic sorting and cultured in serum-free medium were capable of unlimited proliferation and differentiation as cancer stem cells. Flow cytometry showed that the content of CD133+ colon cancer stem cells was 80.3%. Measurements of omentin-1 concentrations on colon cancer stem cells and different time periods suggested that omentin-1 could promote proliferation and inhibit the apoptosis of colon cancer stem cells relative to the passage of time. In addition, the authors showed that the combination of omentin-1 and LY294002 had a synergistic effect on the proliferation activity and inhibition of apoptosis of colon cancer stem cells, which was more expressed than the use of omentin-1 or LY294002 alone [59].

According to Datta et al. [60], omentin-1 expression increases during gastrointestinal infection. Infection of resistant or susceptible BALB/c mice with *Trichuris muris* (a nematode parasite of mice) provokes the expression of several antimicrobial genes, including omentin-1.

### 3.2. Liver Cancer

In vitro experiments have shown that administration of omentin-1 to hepatocellular carcinoma cell cultures increases the expression of p53 protein without increasing TP53 mRNA levels, suggesting that the increase in this protein may be related to its post-translational modification (acetylation). Omentin-1 inhibits p53 deacetylation by inhibiting the action of Sirt1 deacetylase. Incubation of cell cultures with 1 μg/mL omentin-1 for 24 h resulted in a 2.5-fold increase in the Bax/Bcl-2 ratio and an increase in caspase 3 activity [19]. Zhang et al. examined the effects of omentin-1 on two types of human hepatocellular carcinoma cells: HepG2 and HuH-7. The authors noted that omentin-1 (1 and 2 μg/mL) inhibited the proliferation of HepG2 and HuH-7 cells. Omentin-1 stimulation increased p53 and p21 protein levels. Interestingly, omentin-1 did not affect TP53 mRNA levels. Further investigation of the mechanism of action revealed that omentin-1 decreased p53 deacetylation, thereby increasing p53 protein stability. HepG2 cells stimulated with omentin-1 presented an increase in the Bax/Bcl-2 ratio and activation of the caspase-3 pathway. Omentin-1 induced the JNK signaling pathway but not the p38 and ERK1/2 signaling pathways. These findings indicate that omentin-1 may be the therapeutic strategy for hepatocellular carcinoma [19].

### 3.3. Lung Cancer and MPM

Under normal conditions, omentin-1 expression is relatively high in healthy lungs compared with other organs and tissues; however, this changes in lung cancer. Although the mechanism is currently unknown, omentin-1 is thought to play an important role in inducing genomic instability and mutation and promoting inflammatory responses that support cancer development. It has been noted that patients with lung cancer and high plasma omentin-1 levels have a higher survival rate compared with those with low levels. Omentin-1 expression negatively correlates with oncogenic transcription factors (FOXA1, FOXC1, EN1, and ELK4), and there is a limited expression of omentin-1 in lung cancer, regardless of sex, cancer subtype, age, and smoking. Studies of other adipokines did not show significant deviations. Omentin-1 and omentin-2 gene expression is significantly reduced in lung cancer. Differential gene expression analysis identified 82 significantly overlapping downregulated differentially expressed genes (DEGs), including omentin-1, which was associated with a better prognosis for patients with lung cancer. This indicates a possible protective function of omentin-1 in lung cancer and may also explain the paradox between increased BMI and better lung cancer outcomes [61].

MPM is a rare but deadly cancer. Surgical treatment is most effective in the early stages of the disease. Overall, however, the current treatment results for MPM are unsatisfactory. Unfortunately, due to the lack of characteristic clinical symptoms, radiological features, and specific diagnostic markers, MPM is usually diagnosed late. Wali et al. [37] used serial analysis of gene expression (SAGE) to analyse the gene expression pattern of MPM sections with autologous morphologically normal mesothelium. Tumour tissues showed marked overexpression of omentin-1 (>139-fold). Subsequently, they confirmed overexpression of omentin-1 mRNA based on reverse-transcription polymerase chain reaction in four of five resected MPM tumours. They also confirmed omentin-1 protein expression via immunohistochemistry in 28 of 53 tumour samples and via Western blot in 4 of 4 mesothelioma cell lines. Overexpression of omentin-1 in mesothelioma may have potential screening and therapeutic implications [37].

Pleural effusion caused by pleuritis typically contains more omentin-1 than pleural effusion caused by lung cancer. This finding suggests that omentin-1 may be induced in mesothelial cells by inflammatory stimulation, such as by Th2 cytokines, and act as a defense protein in response to airway inflammation, such as in response to asbestos exposure [37]. Patients with MPM have higher levels of omentin-1 than patients with lung cancer and pleural effusion. The mean concentration of omentin-1 in the pleural effusion of patients with MPM is around 3000 ng/mL, while for patients with lung cancer, tuberculosis, and pleuritis, the mean omentin-1 concentrations are around 300, 250, and 650 ng/mL, respectively [62]. To elucidate the mechanism of neoplastic transformation of mesothelial cells, it may be useful to further investigate what kind of stimulation induces omentin-1 expression in mesothelial cells. Currently, mucinous adenocarcinomas can be successfully distinguished from MPM by other clinical methods, but omentin-1 in pleural effusion may be used in the future as a specific diagnostic marker to distinguish epithelioid-type MPM from other cancers due to its specificity and the simplicity of the evaluation [63].

### 3.4. Pancreatic Cancer

Although omentin-1 levels are altered in some cancers, the clinical significance of this change remains unclear in patients with pancreatic cancer. Only a few studies have been conducted. Both colorectal and pancreatic cancer are associated with obesity, metabolic syndrome, and BMI. In the early stages of pancreatitis, there is upregulation of omentin-1, which likely causes insulin resistance and reduces glucose levels [35]. Serum omentin levels of rats with chronic pancreatitis were increased compared with rats with acute pancreatitis and the control groups, so it correlates with the stage of cancer. Karabulut et al. [43] reported that serum omentin-1 levels are elevated in patients with PA (pancreatic adenocarcinoma) and significantly proportional to tumour size, indicating that serum omentin-1 levels may serve as a diagnostic marker in patients with pancreatic cancer. However, the authors did not determine its predictive and prognostic values. Moreover, there was no correlation between serum omentin-1 levels and response to chemotherapy [43]. Kiczmer et al. [64] found significantly increased plasma omentin-1 levels in patients with pancreatic cancer compared with the control group. They found no correlation between the omentin-1 concentration and BMI.

### 3.5. Breast Cancer

Breast cancer is the leading cause of cancer-associated death in women throughout the world. Tahmasebpour et al. [65] observed significantly lower plasma omentin-1 levels in patients with breast cancer compared with healthy controls. In addition, omentin-1 gene expression was significantly reduced in tumour tissues compared with adjacent morphologically normal tissues. Omentin-1 gene expression and serum levels were significantly higher in stage I disease compared with stage II and III disease. In addition, serum omentin-1 levels in patients with p53-positive breast cancer were significantly higher than in patients with p53-negative breast cancer [65]. There were similar observations after analysing 30 postmenopausal patients with breast cancer. The study showed that omentin-1 levels were significantly reduced in patients compared with healthy controls and showed a negative correlation of omentin-1 with BMI in both groups. Panagiotou et al. [53] obtained similar results, indicating that it is likely that anthropometric and metabolic parameters, rather than omentin-1, are the main promoters of carcinogenesis. In addition, the authors noted that serum omentin-1 levels are increased in patients with more aggressive breast cancer based on the Elston–Ellis classification system, which could be explained by the activation of the Akt signaling pathway or inflammatory processes [53].

In a cross-sectional study, Christodoulatos et al. [66] observed an independent negative association between plasma omentin-1 levels and breast cancer incidence in a postmenopausal population, in addition to known breast cancer risk factors as well as metabolic and inflammatory parameters. The plasma omentin-1 concentration was negatively correlated with prevalent cardiometabolic risk factors and positively correlated with the maintenance of a Mediterranean diet. In addition, plasma omentin-1 levels as a biomarker showed similar diagnostic performance to CA15-3 with poorer discriminatory ability in postmenopausal women with obesity [66]. Abas et al. [67] suggest that omentin-1 levels may complement the prognostic information available from classic prognostic factors such as tumor size, lymph node metastasis, and metastasis. In addition, the authors found no significant association between omentin-1 and prognostic factors such as hormone receptors and HER2 (human epidermal growth factor receptor 2) [67].

### 3.6. Gastric Cancer

Based on the high omentin-1 levels reported in colon adenocarcinoma, there has been speculation concerning the involvement of omentin-1 in other gastrointestinal cancers. There are only a few publications on the importance of omentin-1 in gastric cancer, yet the results are significant and clearly indicate the role and importance of omentin-1 in the development and progression of this disease. As we mentioned before, omentin-1 expression increases during gastrointestinal infection [60]. In fact, Zheng et al. [41] showed that omentin-1 expression in normal, healthy gastric mucosa tissue in individuals without a pathological condition—including gastric cancer—is undetectable, and it is expressed in the majority (>70%) of gastric cancer cases. Interestingly, omentin-1 expression was detected only in the cytoplasm of cancer cells and goblet cells of intestinal metaplasia. In samples taken from nearly 200 tested patients, omentin-1 expression was higher in patients with intestinal-type carcinomas than those with diffuse-type carcinomas. Omentin-1 expression correlated positively with tumour differentiation but inversely with the depth of invasion, the presence of lymph nodes, distant metastasis, and clinical stage [41]. The authors indicated that based on univariate and multivariate analyses, omentin-1 is an independent prognostic factor for longer overall survival of patients with gastric cancer, suggesting the role of omentin-1 expression in predicting the outcome of the disease. It is interesting that transfection of the omentin-1 gene conducted by this group of researchers attenuated the proliferation, migration, and invasion of cultured gastric cancer cells in vitro. They speculate that omentin-1 may act as a tumour suppressor in gastric cancer, an eventuality that warrants further research.

Zhang et al. [41] implicated the role of omentin-1 in the aggressiveness of gastric cancer. In 2015, the same team investigated the functions, targets, and, most importantly, the clinical significance of omentin-1 in the progression of gastric cancer [68]. In 90 well-established primary samples from gastric cancer tissues, hepatocyte nuclear factor 4 alpha (HNF4α) expression correlated positively with omentin-1. Importantly, the patients with high omentin-1 and HNF4α expression had a better survival probability. Certainly, more in vitro as well as in vivo experiments are needed to determine the mechanisms underlying omentin-1 induction in gastric cancer cells and the activity of omentin-1 as a therapeutic target for patients with gastric cancer.

### 3.7. Ovarian Cancer

Ovarian cancer (OC) is the most lethal gynaecologic malignancy that metastasizes commonly to the well-vascularized omentum, covered by a layer of mesothelial cells. These cells play an important role in transporting fluid and cells through the serous cavities, presenting antigens, and controlling inflammation or tissue repair when needed [69].

In 2020, Au-Yeung et al. compiled a large amount of data on how ovarian cancer cells are capable of downregulating omentin-1 [70]. Their studies showed that omentin levels are reduced in patients diagnosed with neoplasia compared to patients with benign gynecological lesions, as well as in healthy women samples. They discovered the molecular mechanism by which omentin-1 suppresses the malignant phenotypes of OC cells. OC cells can suppress omentin-1 expression in mesothelial cells located in visceral adipose tissue for cancer cell proliferation and metastasis. Adipocytes in TME (via omentin) are capable of insulin-dependent glucose uptake—which results in local glucose reduction required by OC cancer cells (as well as other types of cancer cells) for cellular metabolism. The result of such action is the inhibition of tumour growth. Finally, they evaluated the efficacy of omentin-1 as a therapeutic agent for OC treatment. They conducted a number of studies in an in vivo model by injecting immunocompetent C57BL/6 mice with OC IG10 intraperitoneally. Data confirmed that recombinant mouse omentin-1 suppressed the migration rate and downregulated MMP1 protein expression in IG10 in vitro [70].

Some markers commonly used in the diagnosis of ovarian cancer, such as CA125 and HE4, are also used in the screening and diagnosis of other malignancies, including endometrial cancer. It seems that the participation of adipokines, including omentin-1, in the development of OC should be significant and may be described as the next biomarker of this disease [71]. Data on this subject are limited, which is why we are still waiting for further results that will enrich our knowledge.

### 3.8. Endometrial Cancer

Diabetes and insulin resistance are both known risk factors for endometrial cancer (EC), one of the most often diagnosed gynecological neoplasms. Soliman et al. [72] studied the effect of a short course of metformin in women with newly diagnosed EC and showed that metformin treatment caused relevant serum and molecular changes, including decreased significantly levels of omentin-1, which was confirmed later by Yates et al. [73]. They also noted that the inhibitory effect of metformin is not only based on lowering serum omentin levels but also affects the PI3K/AKT/mTOR signaling pathway through AMP activation and phosphorylation of live kinase B1 (LKB1). It is worth noting that this pathway is a key element in the activation of obesity-associated endometrial cancer cells [71,72].

An interesting study was carried out by Cymbaluk-Płoska et al. [74]. They investigated the association between serum concentrations of adipose tissue metabolism products (four selected adipokines, including omentin-1) and the diagnostics and prognosis of endometrial cancer. The results showed that the concentration of omentin-1 during the development of EC is significantly reduced compared to physiological serum levels and correlates with the stage of EC (the concentration decreases at each stage of EC progression). Interestingly, lower values of omentin-1 (and vaspin) concentrations were also presented in EC patients with lymphatic vessel invasion, lymph node metastases, and deep endometrial infiltration [74]. In the later study of this research team [75], they showed a dependent impact of five factors, including omentin (secreted by the adipose tissue and involved in the regulation of glucose and lipid metabolism), as well as factors linked with oxidative stress responsible for inflammatory processes and angiogenesis, such as IL-1β, IL-6, IL-8, TNFα, VEGF, and FGFs. They assessed their importance in the diagnosis and prognosis of EC.

Summarizing, all reported results showed significantly reduced serum or plasma omentin-1 concentrations among patients with endometrial cancer (EC).

### 3.9. Prostate Cancer

For the first time, the relationship between omentin level and prostate cancer (PC) progression was investigated in 2014 by Uyeturk et al., which is surprising because PC is the second most frequent cancer diagnosis made in men worldwide. They assessed omentin levels in 50 prostate cancer patients in a matched case–control study, and the results indicated that the median omentin concentration in PC patients was significantly higher compared to those with benign prostatic hyperplasia (BPH) [39]. In fact, since then, plasma or serum omentin-1 levels in PC have rarely been explored. Fryczkowski et al. [76] measured the relationship between circulating levels of PSA, hepatocyte growth factor (HGF), vascular endothelial growth factor (VEGF), and omentin-1 (as one of the adipokine) in serum obtained from BPH patients and PC patients. The results showed that not only omentin-1 but also growth factors—HGF and VEGF—concentrations were significantly higher in the PC than in the BPH group. Studies by He et al. indicated that lower serum omentin-1 levels were associated with mRNA overexpression of IL-8 and IL-18 in the BPH prostatic cells and higher prostate volume [77].

In 2019, Zhou et al. confirmed the thesis that omentin is overexpressed in PC patients compared to healthy individuals [78]. Additionally, the circulating omentin-1 concentrations positively correlated with obesity-related markers, such as body mass index (BMI) and waist–hip ratio (WHR), as well as prostate-specific antigen (PSA)—one of the basic markers determined in prostate cancer screening. These data indicated that omentin-1 might have a potential role in the development of PC, although they did not confirm in their research a significant relationship between the PC tumour grades and the omentin-1 level [78]. Obesity is considered one of the PC risk factors [79], which was also confirmed in meta-analyses [7,80]. There is an association between the omentin-1 level and BMI and obesity in patients with PC; this is not surprising and confirms the observations in other types of cancers.

PC has also been linked to androgen exposure, so androgen deprivation therapy was often recommended for PC patients, although research has shown that lowering endogenous testosterone does not protect against PC development, and testosterone replacement therapy does not increase PC risk [81]. Importantly, the data show, in fact, that low serum testosterone levels in PC patients may be correlated with worse prognosis and more aggressive type of cancer [82]. Due to the dependence of secreted adipokines on the amount of adipose tissue (the accumulation of which is controlled by hormones, such as androgens and estrogens), it was natural to check whether this sex hormone affects the expression and level of omentin-1, as one of the adipokines secreted by fat. Since prostate activity is controlled by estrogens, androgens, and sex hormone-binding globulin (SHBG) responsible for the transport of sex hormones to their target cells, the correlation of omentin, sex steroids, SHBG, as well as metabolic syndrome and age was investigated in PC patients by Borowski & Siemińska [83]. In their results, they showed that omentin levels did not correlate with estradiol or testosterone concentrations but were dependent on the testosterone/SHBG ratio and had a positive correlation with SHBG. The authors indicate that serum omentin levels may be a diagnostic marker in prostate cancer patients, although omentin has not been shown to be associated with an increased risk of developing metabolic syndrome in men with PC.

All data presented in the manuscript regarding the role of omentin-1 in various types of cancer cells are summarized in Table 1.

## 4. Conclusions

Omentin-1 is an adipokine released by adipose tissue. It performs a variety of functions in numerous metabolic processes, including carcinogenesis. A number of studies suggest that it is involved in carcinogenesis, although knowledge is incomplete. Omentin-1 has anti-apoptotic activity but can also enhance the apoptosis of cancer cells. This adipokine can affect angiogenesis, endothelial function, and the expression of adhesion molecules. Studies are currently being conducted on the potential of omentin-1 as a marker of cancer progression. These studies aim to clarify whether the omentin-1 concentration depends on the type of cancer, its stage, and the severity of the metastatic process and whether it can be an indicator of the effectiveness of the anti-cancer therapy used. Elevated plasma omentin-1 levels may result in part from the tumour or the degree of weight loss associated with cancer. Although the function of omentin-1 in the cancers described is still not completely understood, some of the evidence presented in this review suggests that local tumour omentin-1 levels are a good prognostic indicator. Additional research is needed to determine whether the change in omentin-1 levels is a cause or effect of cancer development, and elucidating this causal relationship will undoubtedly contribute to clarifying the correlation between obesity and cancer. Currently, there is also a need for additional research to determine the diagnostic value of measuring omentin-1 levels. Studies are also needed to determine the interaction between omentin-1 and other adipokines and how omentin-1 exerts its regulatory roles. The effects of various diseases on omentin-1 concentrations also need to be clarified. Research is also needed on the use of omentin in the treatment of cancers. Thus, there is a need for multicentre clinical studies to assess the potential importance of omentin-1 as a marker of cancer progression and the use of this adipokine in anti-cancer therapy.

## Figures and Tables

**Table 1:** Role of omentin-1 in cancer cell biology.

Type of Cancer	Expression in Tested Samples	Effect in Cancer Cells	References
Colorectal cancer	↑	Localized in the cytoplasm;	[20,60]
		Omentin-1/TMEM207 described as a prognostic biomarker;	
		↑ in gastrointestinal infection.	
Liver cancer	No data	↑ protein levels of p53 and p21; ↓ p53 deacetylation and ↑ the stability of p53 protein;	[19]
		↑ bax/bcl-2 protein ratio; activated the caspase-3 signaling pathway; triggered JNK but not p38 and ERK1/2 signaling pathways.	
Lung cancer	↑	Negatively correlated with oncogenic transcription factors (FOXA1, FOXC1, EN1 and ELK4).	[61,62,63]
Pancreatic cancer	↑	↓ glucose levels;	[35,43]
		↑ insulin resistance.	
Gastric cancer	↑	↑ the levels of hepatocyte nuclear factor 4 alpha (HNF4α), which suppresses the progression of gastric cancer; ↓ functions and activity of nuclear factor-kappa B (NF-kB); ↓ PI3K/Akt signaling pathway.	[41,68]
Breast cancer	↓	Similar diagnostic value to CA15-3.	[66]
Ovarian Cancer	↓	Adipocytes in TME are capable of insulin-dependent glucose uptake via omentin; Suppressed OC growth by increasing adipocytes’ glucose uptake through GLUT4 upregulation.	[70]
Endometrial Cancer	↓	Metformin reduced omentin levels via PI3K/AKT pathway.	[72,74]
Prostate Cancer	↑	Associated with ↑ prostate volume and ↑ IL-8 and IL-18 expression levels in BPH prostatic cells. Omentin-1 described as a diagnostic indicator; Related to the testosterone/SHBG ratio.	[75,77]
